# Interventions for cognitive symptoms of dementia ‐ and the Lancet commission view on anti‐amyloid antibodies

**DOI:** 10.1002/alz.085066

**Published:** 2025-01-09

**Authors:** Geir Selbaek

**Affiliations:** ^1^ Faculty of Medicine, University of Oslo, Oslo Norway

## Abstract

**Background:**

While symptomatic treatment for Alzheimer’s disease has been available for three decades disease modifying treatment does not exist. New drug treatments, known as immunotherapies, which remove amyloid from the brain offer hope, but the clinical significance remains uncertain.

**Method:**

Systematic reviews of the literature, expert consensus, and information from clinical environments.

**Result:**

Cholinesterase inhibitors (ChEI) and memantine have repeatedly demonstrated a modest symptomatic effect on cognition and function in clinical trials and long‐term observational studies indicate that these drugs may lower mortality. They are readily available, relatively affordable, have few side‐effects and should be offered to patients with Alzheimer’s disease or Lewy Body dementias.

Treatment with the monoclonal antibodies lecanemab and donanemab consistently shows an effect on clinical measures, such as cognition, function, and quality of life. However, the effect size is small, and the results may not generalize to groups that are poorly represented in the clinical trials. Risk of serious adverse events, such as brain haemorrhages, may restrict the population eligible for treatment. Lecanemab has been granted marketing approval in USA, Japan and China and awaits decision in European countries, expected first half of 2024. Before implementation several issues need to be addressed. More people will need a more precise and biomarker‐based diagnosis at an earlier disease stage. The total treatment cost is huge. In addition to medication cost more resources will be needed for diagnostic testing, treatment administration and surveillance/monitoring of side effects. At present, very few countries can meet these requirements.

**Conclusion:**

The advent of monoclonal antibodies represents a major scientific breakthrough in the quest to combat Alzheimer’s disease and might offer hope to people with the disease and their closest caregivers. Unfortunately, so far, the limited clinical gain may not offset treatment cost and risk of adverse events. Long‐term efficacy data and results from trials with treatment initiated at an earlier disease stage are needed to determine the meaningfulness of these new treatments. In parallel, other treatment options targeting tau protein, inflammatory pathways or metabolic dysfunction should be investigated further to offer people with Alzheimer’s disease and related dementias a personalized treatment regimen.

